# Standard versus simplified radiofrequency ablation protocol for Barrett’s esophagus: comparative analysis of the whole treatment pathway

**DOI:** 10.1055/a-1005-6331

**Published:** 2020-01-22

**Authors:** Wei Keith Tan, Krish Ragunath, Jonathan R. White, Jose Santiago, Jacobo Ortiz Fernandez-Sordo, Mirela Pana, Bincy Alias, Andreas V. Hadjinicolaou, Vijay Sujendran, Massimiliano di Pietro

**Affiliations:** 1 MRC Cancer unit, University of Cambridge, Cambridge, UK; 2 Department of Gastroenterology, Addenbrookes Hospital, Cambridge, UK; 3 NIHR Nottingham Biomedical Research Centre, Nottingham University Hospitals NHS Trust and The University of Nottingham, Nottingham, UK; 4 Nottingham Digestive Diseases Centre, The University of Nottingham, Nottingham, UK; 5 Department of Surgery, Cambridge University Hospital NHS Foundation Trust, Cambridge, UK

## Abstract

**Background and study aims** The standard radiofrequency ablation (RFA) protocol for Barrett’s esophagus (BE) encompasses an intermediary cleaning phase between two ablation sessions. A simplified protocol omitting the cleaning phase is less labor-intensive but equally effective in studies based on single ablation procedures. The aim of this study was to compare efficacy and safety of the standard and simplified RFA protocols for the whole treatment pathway for BE, including both circumferential and focal devices.

**Patients and methods** We performed a retrospective analysis of prospectively collected data on patients receiving RFA between January 2007 and August 2017 at two institutions. Outcomes assessed were: 1) complete remission of dysplasia (CR-D) and intestinal metaplasia (CR-IM) at 18 months; and 2) rate of esophageal strictures.

**Results** One hundred forty-five patients were included of whom 73 patients received the standard and 72 patients received the simplified protocol. CR-D was achieved in 94.5 % and 95.8 % of patients receiving the standard and simplified protocol, respectively ( *P* = 0.71). CR-IM was achieved in 84.9 % and 77.8 % of patients treated with the standard and simplified protocol, respectively ( *P* = 0.27). Strictures were significantly more common among patients who received the simplified protocol (12.5 %) compared to the standard protocol (1.4 %; *P* = 0.008). The median number of esophageal dilations was one.

**Conclusion** The simplified RFA protocol is as effective as the standard protocol in eradicating BE but carries a higher risk of strictures. This needs to be taken into account, particularly in patients with higher pretreatment risk of strictures, such as those with esophageal narrowing from previous endoscopic mucosal resection (EMR).

## Introduction

Barrett’s esophagus (BE) is a premalignant condition characterized by the replacement of the native esophageal squamous epithelium by columnar epithelial with intestinal metaplasia (IM) 1 2 . BE predisposes to esophageal adenocarcinoma, an aggressive cancer with an overall 5-year survival of only 17 % 3 . Radiofrequency ablation (RFA) has been established as a safe, effective and durable treatment for flat, dysplastic BE, 4 5 6 7 . RFA is delivered using the BARRX system, which comprises a circumferential ablation device (BARRX 360) and focal ablation devices (BARRX 60, BARRX 90, BARRX Ultra or through-the-scope device).

RFA protocol varies between institutions. The most commonly applied protocol incorporates a cleaning phase, whereby debris from the ablated mucosa (one ablation at 12 J/cm ^2^ for circumferential RFA or two ablations at 15 J/cm ^2^ for focal RFA) is removed with cleaning devices, followed by a second round of ablation with an energy setting identical to the first 8 9 10 11 . This regimen is referred to as the “standard protocol.” Although effective, this regimen is tedious and time-consuming and requires multiple intubations.

To reduce procedural time and increase tolerability of RFA, van Vilsteren et al performed two randomized studies to evaluate the efficacy of a “simplified protocol” which omits the cleaning phase. In the first study, circumferential ablations with standard (1 × 12 J/cm ^2^ -clean-1 × 12 J/cm ^2^ ) versus simplified (2 × 12 J/cm ^2^ ) protocol without cleaning were found to be equally effective based on subjective assessment of the percentage of BE regression at follow-up 12 . In the second study, investigators randomized single islands of Barrett’s mucosa to either standard (2 × 15 J/cm ^2^ -clean-2 × 15 J/cm ^2^ ) or simplified (3 × 15 J/cm ^2^ -no clean) focal protocols and found no significant difference between the two 13 .

Following this evidence, many centers have adopted the simplified protocol as it is easier and better tolerated by patients, however, the randomized studies only referred to efficacy and safety of individual treatments and not the overall treatment pathway. A retrospective study of 83 patients showed that multiple treatments over time with the simplified protocol (3 × 15 J/cm ^2^ -no clean) achieved CR-D and CR-IM in 94 % and 87% of patients, respectively, but were complicated by strictures in 11 % of patients, who required a median of two dilation sessions 14 . However, there remain concerns that consecutive ablations may cause deeper damage to the esophageal mucosa, possibly due to build-up of radiofrequency energy, which may cause strictures. The dosimetry of 3 × 15 J/cm ^2^ for the simplified protocol was thought to be too aggressive and following a consensus meeting in Amsterdam, a lower dose of 3 × 12 J/cm ^2^ was suggested. Therefore, most European centers have now adopted the lower 3 × 12 J/cm ^2^ dose for focal ablations. Currently there is still no universally defined regimen for RFA for dysplastic BE and data are lacking on efficacy and safety of the two protocols when they are assessed over the course of the entire RFA treatment pathway. The aim of this study was to investigate efficacy and safety of the standard and simplified protocols for treatment of BE including both circumferential and focal treatments. We hypothesize that the simplified protocol will be as effective as the standard protocol in achieving CR-D and CR-IM but may carry a higher risk of strictures.

## Patients and methods

We performed a retrospective analysis of prospectively collected data among patients who received RFA treatment for dysplastic BE between July 2007 to August 2017 at two UK tertiary referral centers.

### Inclusion criteria

Patients were eligible if they had flat BE with low-grade dysplasia (LGD) or high-grade dysplasia (HGD) and residual BE post-endoscopic mucosal resection (EMR) for low-risk BE-related early cancer. BE was defined as presence of endoscopically visible columnar epithelial with biopsy confirming IM. For LGD, RFA was performed once LGD was confirmed by two gastrointestinal pathologists on two separate endoscopies. For flat HGD, RFA was performed after histological confirmation from a single endoscopy by two gastrointestinal pathologists. Low-risk intramucosal carcinoma (IMC) was defined as well-differentiated IMC (T1b sm1, within 500 um of the submucosa) without lymphovascular invasion. Dysplasia was classified according to the Vienna Classification 15 . Patients were categorized according to the highest grade of dysplasia detected during their endoscopic management. Patients who had prior EMR for nodular BE were eligible for inclusion if they underwent subsequent RFA.

### Endoscopic procedures

Patients were scoped with GIF-FQ260Z, GIF-H290Z or GIF-HQ290 endoscopes (Olympus, Tokyo). BE was graded according to the Prague classification 16 . Random quadrantic biopsies were taken with adherence to the Seattle protocol 17 . Visible lesions seen during endoscopy were classified according to the Paris classification 18 . Superficial lesions (type 0) were treated with EMR for full histopathological staging. RFA was then performed on the residual flat BE at least 6 weeks post-EMR.

### Radiofrequency ablation protocol

RFA was performed using the BARRX ablation devices (Medtronic; Sunnyvale, California, United States). RFA was repeated at roughly 3-month intervals. Circumferential ablation was performed with an appropriate-size balloon selected after estimation of esophageal diameter using a sizing balloon. Sizing and treatment balloons were introduced blindly over a guidewire. In patients with previous EMR, a smaller balloon was used as per manufacturer’s recommendation.

Circumferential ablation with the standard protocol was performed for each segment of the BE starting from the proximal end with one ablation at 12 J/cm ^2^ , followed by cleaning of mucosal slough with esophageal cap, patient extubation, cleaning of ablation device with wet gauze, and finally a second ablation at 12 J/cm ^2^ (1 × 12 J/cm ^2^ -clean-1 × 12 J/cm ^2^ ). Focal ablation using the standard protocol was performed with two ablations at 15 J/cm ^2^ , until coverage of the whole segment, followed by cleaning of the mucosal slough with the focal device, patient extubation, cleaning of the RFA device with wet gauze and finally further treatment with two ablations at 15 J/cm ^2^ per BE area (2 × 15 J/cm ^2^ -clean-2 × 15 J/cm ^2^ ).

Circumferential ablation using the simplified protocol was performed with two consecutive ablations at 12 J/cm ^2^ without cleaning (2 × 12 J/cm ^2^ -no clean) for each segment of the BE until coverage of the whole BE area. Focal ablations were performed with three consecutive ablations at 12 J/cm ^2^ without cleaning (3 × 12 J/cm ^2^ -no clean). Focal ablations were performed using the BARRX 60, BARRX 90, BARRX Ultra and BARRX Through-the-scope (TTS) devices.

During follow-up, argon plasma coagulation was performed at the discretion of the endoscopist to ablate small BE-islands. Uptake of the simplified protocol varied between the institutions with Cambridge adopting the simplified protocol from November 2012, while Nottingham adopted the simplified protocol in January 2015. This change in practice led to some patients receiving a hybrid protocol with the standard protocol during early treatments and the simplified protocol during later visits. In this study, we only included patients who were treated exclusively with either the standard or simplified protocols. We also excluded patients who were treated with the more recent BARRX Express device, which does not require sizing of the esophageal lumen. These cases were excluded due to lack of agreement among users about the optimal protocol to be used, and because of the variation in practice even within the same endoscopy unit.

### Endoscopic follow-up

The interval between follow-up post-RFA was dependent on baseline histology. Patients with LGD were seen at 6 months following complete pathological response and then at 12 months and every 2 years thereafter. Patients with HGD or IMC were seen at 3 months following complete pathological response, and then at 6 months, and annually thereafter. At follow-up, white-light and narrow-band imaging were used to inspect the gastroesophageal junction (GEJ) to identify BE islands. Quadrantic biopsies were taken at the GEJ to detect IM or dysplasia and every 2 cm within the neo-squamous epithelium to detect buried IM. If IM was found at the GEJ at the first post-RFA follow-up, circumferential ablation of the GEJ was performed with focal BARRX device. Focal IM in a single GEJ biopsy at the second or subsequent endoscopic follow-up was not considered BE recurrence and was not an indication for repeat RFA.

### Outcome measures

Outcomes measure were: 1) complete remission of dysplasia (CR-D) and complete remission of IM (CR-IM) at 18 months; and 2) rate of esophageal strictures. CR-D was defined as eradication of dysplasia within 18 months, but with persistent columnar-lined epithelium with IM. CR-IM was defined as complete eradication of both dysplasia and IM. Complete remission of dysplasia or IM was defined as absence of dysplasia or IM on two consecutive biopsies from the last RFA session. We also assessed the rate of strictures post-RFA. Stricture was defined as symptomatic esophageal narrowing requiring dilation to improve symptoms or to allow endoscope passage. Asymptomatic narrowing passed by the endoscope was not considered strictures. Patients were only considered to have an RFA-related stricture if the stricture developed after commencing RFA. Patients who had previous stricture secondary to prior-EMR were not considered to have a stricture, unless the stricture recurred after RFA. Esophageal dilation was performed with a through-the-scope device with the aim of providing symptomatic relief rather than achieving a specific size of the esophagus. The size of the dilatation balloon was selected based on the estimated diameter of the esophagus. If required, open biopsy forceps were used to size the stricture. We also assessed whether age, gender, maximum length of BE, prior-EMR, extent of prior-EMR, baseline histology, type of RFA protocol, and different ablation devices were predictors of stricture formation. For patients who received prior-EMR, the number of EMR specimens performed during a single session was recorded as a continuous variable and acted as a surrogate for the extent of prior-EMR.

### Statistical analyses

Normality testing was performed using the Shapiro-Wilk test. For descriptive statistics, mean (± standard deviation, SD) was used for parametric variables and median (interquartile range, IQR) was used for non-parametric variables. Independent student’s *t* -test and Mann-Whitney test were used to compare means between groups. Rates of CR-D, CR-IM and strictures between groups were compared using Chi-squared test. Binary logistic regression was used to assess for predictors of stricture formation. *P* < 0.05 was considered statistically significant. Statistical analysis was performed using SPSS (version 24; Armonk, New York, United States, IBM Corp).

## Results

### Patient characteristics

**Fig. 1:**
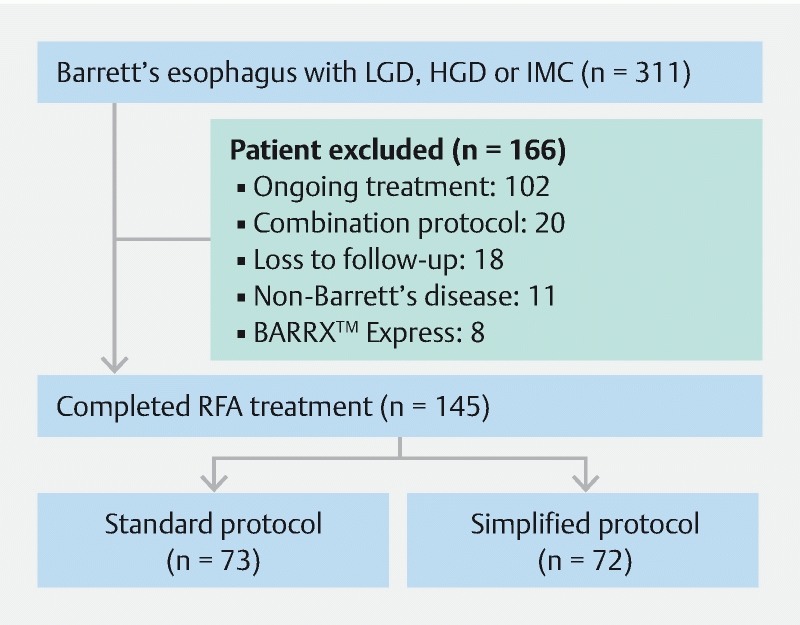
Comparative analysis of radiofrequency ablation (RFA) for standard versus simplified protocol: study flowchart.

In total, 311 patients underwent RFA between July 2007 and August 2017. Of them, 166 patients were excluded due to ongoing RFA treatment (n = 102), combination protocol (n = 20), loss to follow-up (n = 18), RFA for non-Barrett’s disease (n = 11), BARRX Express device used (n = 8), treatment interrupted because of other co-morbidities (n = 6) and patient refusal of further treatment (n = 1) ( Fig. 1 ). A total of 145 patients (97 from Cambridge and 48 from Nottingham) completed the 18-month RFA protocol and were included in our analysis. Of the 145 patients, 73 were exclusively treated with the standard protocol, while 72 patients received the simplified protocol. Mean age was 66.6 years and males (86.2 %) were the predominant gender. The median length of BE was 4 cm (IQR 4.0 cm) and 61.4 % of patients received prior-EMR. For patients who received prior-EMR, the median number of EMR specimens resected (a surrogate for extent of prior-EMR) was 3 (IQR 2). Five cases had missing data on the extent of EMR ( Table 1 ).

**Table 1:** Patient baseline characteristics stratified by different RFA protocols.

Variable	Standard protocol	Simplified protocol	All patients	*P* value
Number of patients	73	72	145	–
Age, mean (SD)	66.3 (9.0)	67.0 (9.7)	66.6 (9.3)	0.66 1
Gender (% males)	86.3	86.2	86.2	0.97 2
BE length, M value (median, IQR)	5.0 (5.0)	4.0 (4.0)	4.0 (4.0)	0.35 3
Prior EMR, n (%)	47 (64.4)	42 (58.3)	89 (61.4)	0.45 2
Extent of prior EMR 4 (median, IQR)	2 (2)	3 (3)	3 (2)	0.12 3
Number of RFA (median IQR)	3 (1)	2 (1)	2 (1)	0.17 3
Grade of dysplasia, n (%)	–	–	–	0.082 2
LGD	7 (9.6)	13 (18.1)	20 (13.8)	
HGD	27 (37.0)	33 (45.8)	60 (41.4)	
IMC	39 (53.4)	26 (36.1)	65 (44.8)	

SD, standard deviation; IQR, interquartile range; lGD, low-grade dysplasia; HGD, high-grade dysplasia; IMC, intramucosal cancer; EMR; endoscopic mucosal resection

1 Studentʼs *t* test

2 Chi-square test

3 Mann-Whitney test

4 The extent of prior EMR was recorded as the number of EMR resections performed during a single session. The number of EMR resections was then used as a surrogate for the extent of prior EMR.

When stratified by protocol type (standard vs simplified), there was no significant difference between the groups in terms of age, gender ratio, BE maximum length, rate of rescue EMR and number of RFA sessions within 18 months. There was no significant difference between the groups in terms of grade of dysplasia, however, the number of LGD cases in the standard protocol group was smaller, in keeping with the fact that RFA for LGD was only recently approved by the British Society of Gastroenterology 19 20 .

### RFA outcomes at protocol endpoint

At protocol endpoint of 18 months, 95.2 % and 81.4 % of patients achieved CR-D and CR-IM, respectively, with a median of two ablations. When stratified to different RFA protocols, there was no significant difference in CR-D outcomes between the standard (94.5 %) and simplified (95.8 %) protocol ( *P* = 0.71). Similarly, there was no significant difference in CR-IM between the standard (84.9 %) and simplified (77.8 %) protocol ( *P* = 0.27). Twelve patients (8.3 %) developed visible nodular lesions during follow-up and required rescue EMR. Among them, only two patients had their BE upstaged. One patient had RFA performed for LGD but required rescue EMR 6 months later, which showed HGD. This patient achieved CR-IM at the end of the protocol. Another patient with baseline HGD required rescue EMR, which showed upstaging of disease to IMC. This patient did not achieve CR-D at the protocol endpoint. The remaining 10 patients who received rescue EMR did not have their disease upstaged ( Table 2 ).

**Table 2:** Characteristics of patients who required rescue-EMR.

Case	RFA protocol	Baseline BE histology	Rescue EMR histology	Development of stricture at end of study
1	Standard	HGD	HGD	No
2	Standard	IMC	Squamous	No
3	Standard	LGD	HGD	No
4	Standard	IMC	LGD	Yes 1
5	Standard	IMC	HGD	No
6	Standard	IMC	LGD	No
7	Standard	LGD	LGD	No
8	Simplified	HGD	NDBE	No
9	Simplified	HGD	IMC	No
10	Simplified	IMC	IMC	No
11	Simplified	HGD	HGD	No
12	Simplified	HGD	NDBE	No

EMR, endoscopic mucosal resection; RFA, radiofrequency ablation; BE, Barrett’s esophagus; NDBE, non-dysplastic Barrett’s esophagus; LGD, low-grade dysplasia; HGD, high-grade dysplasia; IMC, intramucosal carcinoma

1 Stricture developed after RFA and prior to receiving rescue EMR

### Complications from RFA

Ten patients (6.9 %, 95 % CI 2.7 – 11.1) developed esophageal strictures. One patient in the standard protocol group developed a stricture (1.4 %, 95 % CI 0 – 4.1) compared to nine patients (12.5 %, 95 % CI 4.7 – 20.3) in the simplified protocol group ( *P* = 0.008) ( Table 3 ). A median of one dilation session was required to treat the stenosis (range 1 – 7). Two patients required more than one dilation, one of whom required two dilation sessions and the other required seven dilation sessions. Among the 12 patients who required rescue-EMR, only one patient developed a stricture, which occurred after the first RFA (BARRX 360) and prior to the rescue EMR.

**Table 3:** Comparative outcomes of RFA with standard versus simplified protocol.

Variable	Standard protocol (n = 73)	Simplified protocol (n = 72)	All patients (n = 145)	*P* value
CR-D, % (95 % CI)	94.5 (89.2 – 99.9)	95.8 (91.1 – 100)	95.2 (91.6 – 98.7)	0.71 1
CR-IM, % (95 % CI)	84.9 (76.5 – 93.3)	77.8 (67.9 – 87.6)	81.4 (75.0 – 87.8)	0.27 1
Rescue EMR, % (95 % CI)	9.6 (2.7 – 16.5)	6.9 (0.9 – 13.0)	8.3 (3.7 – 12.8)	0.56 1
Strictures, % (95 % CI)	1.4 (0 – 4.1)	12.5 (4.7 – 20.3)	6.9 (2.7 – 11.1)	0.008 1

CR-D, complete remission of dysplasia; CR-IM, complete remission of intestinal metaplasia; CI, confidence interval; EMR, endoscopic mucosal resection

1 Chi-square test

We also looked for clinical predictors of stricture following RFA. We assessed age, gender, maximum length of BE, prior-EMR, extent of prior-EMR, baseline histology and type of RFA protocol as predictors of stricture formation post-RFA. Logistic regression showed that protocol type was the only significant predictor of stricture. The simplified protocol was associated with 11 times greater odds of developing strictures compared to the standard protocol (OR: 11.0, 95 % CI 1.27 – 83.45, *P* = 0.029). Extent of prior EMR had no association with risk of developing strictures (OR 1.09, 95 % CI 0.76 – 1.57, *P* = 0.63). With regard to the type of device used, we considered a total of 371 ablations performed. Type of ablation device (BARRX 360, BARRX 90, BARRX-TTS, BARRX Ultra and BARRX 60) also was not associated with risk of stricture ( *P* = 0.78) ( Table 4 ).

**Table 4:** Comparative outcomes of RFA for different devices.

Ablation devices	Number of strictures	*P* value
Total BARRX ablations (n = 371)	10	0.78 1
BARRX 360 (n = 75)	3	
BARRX 90 (n = 239)	5	
BARRX TTS (n = 13)	0	
BARRX Ultra (n = 23)	1	
BARRX 60 (n = 21)	1	

TTS, through-the-scope

1 Chi-squared test

We observed serious adverse events (SAE) in two patients treated with the standard protocol. One patient developed symptomatic atrial fibrillation within 36 hours after two ablations (one with circumferential and one with focal device) and required pharmacological cardioversion. Another patient was admitted within 48 hours after RFA with severe chest pain and dysphagia, which was not related to esophageal stricture and was managed conservatively with analgesia and intravenous fluids. We also observed one SAE in a patient treated with the simplified protocol who developed melena 48 hours after RFA. Gastroscopy showed no evidence of active bleeding and he was discharged after observation for 24 hours.

## Discussion

RFA for BE has been widely available for over a decade, however, there is geographical variation in the protocol used in clinical practice and there remains uncertainty about the optimal energy dosimetry. Early studies of focal RFA used a regimen with a single ablation, followed by cleaning of the ablation zone and subsequent application of a single ablation 10 11 . However, due to lack of efficacy, the number of ablations were doubled (2 × 12 J/cm ^2^ -cleaning-2 × 12 J/cm ^2^ ) resulting in a higher rate of BE eradication 11 . However, subsequent RFA studies on HGD and EAC, which assessed higher-energy dosimetry, showed that a (2 × 15 J/cm ^2^ -cleaning-2 × 15 J/cm ^2^ ) resulted in better efficacy without compromising the complication rate 10 . This higher dosimetry was then incorporated into European practices, however, the 2 x12 J/cm ^2^ -cleaning-2 × 12 J/cm ^2^ was still favored in US-based studies for focal ablation 14 .

It was then hypothesized that the intermediary cleaning phase using the esophageal cap could inherently lead to mucosal edema, decreasing efficacy of the second cycle of ablations. In two randomized trials based on single RFA procedures, Van Vilsteren et al showed that the simplified protocol without cleaning for both circumferential or focal RFA was equally effective as the standard protocol 12 13 . Although omitting the cleaning phase did not reduce RFA efficacy, it allowed for a shorter procedure time. However, these studies only compared ablation outcomes of a single treatment session, hence the overall safety of the entire treatment pathway could not be assessed.

Kunzli et al then compared the efficacy of the simplified protocol using focal ablation device only (BARRX 90) for the whole treatment pathway. Once again, the simplified protocol was effective in BE eradication compared to historical literature, but induced esophageal strictures in 11 % patients. Although the median number of dilations required was two, three patients required at least eight endoscopic dilations 14 . More recently, Pouw et al conducted a randomized non-inferiority trial comparing the efficacy of the simplified (3 × 12 J/cm ^2^ ) and standard (2 × 15 J/cm ^2^ -cleaning-2 × 15 J/cm ^2^ ) regimen using focal RFA for dysplastic BE with a pre-specified non-inferiority margin of –15 % and showed that the simplified protocol was non-inferior to the standard protocol in achieving BE surface regression after two ablation sessions 21 . Esophageal stenosis, which was one of the secondary outcomes in this study, showed no difference between the simplified and standard protocol (9 % vs 11 %, *P* = 1) 21 . The latter two studies only assessed focal ablations using the BARRX 90 for ablation of BE islands and tongues. Eligibility criteria allowed for patients with preceding circumferential ablation using BARRX 360, however, it was not specified whether patients who receive circumferential ablation were treated with the standard of simplified protocol, or combination of both 14 . In our study, we included patients who were exclusively treated with either the simplified or standard protocol for the whole treatment pathway of BE, including both circumferential and focal ablation as clinically indicated. Our results showed that outcomes of CR-D and CR-IM were similar with the standard and simplified protocols.

However, we found a higher number of strictures in patients treated with the simplified protocol. The rate of strictures was significantly higher in the simplified protocol (12.5 %) compared to the standard protocol (1.4 %). This could be secondary to the “stacking” of energy with consecutive ablations which penetrate deeper into the esophageal wall, leading to more extensive damage and stricture development. Although the randomized trial by Pouw et al showed no significant difference between the rate of esophageal strictures, that study was powered to detect a difference between BE surface regression, and not to detect a difference between the rate of strictures. Further, this study also did not include patients undergoing circumferential ablation 21 .

The higher stricture rate for the simplified protocol needs to be considered in cases that have a higher pretreatment probability of stricture, such as those with preexisting stricture from previous EMR who could have already been treated with dilatation. For these cases, the standard ablation protocol could be used. The ablation device used in our study was the older-generation BARRX 360 circumferential device with separate sizing balloon, which is no longer commercially available and has been replaced by the BARRX 360 Express catheter. The findings from our study have potential implications for the new BARRX 360 Express device, which is longer and more compliant to the esophagus than the BARRX 360 circumferential device and has been shown to correlate in early clinical experience to a high rate of strictures 22 23 . Our data would suggest caution in selecting the simplified protocol without cleaning phase when using the BARRX 360 Express device. None of the patients included in our study were treated with the BARRX 360 express catheter. A randomized trial is currently ongoing to assess the comparative efficacy and safety of standard and simplified protocol for BARRX 360 express 22 .

In our current study, we selected a more liberal RFA protocol duration of 18 months compared to 12 months for the UK registry, and in other prospective studies, as we felt that this more accurately reflects the high demands and prolonged waiting list for endoscopy seen in large UK tertiary hospitals 4 24 . Although this could lead to overestimation of CR-D and CR-IM, in this study, the mean number of ablations over 18 months (2.6) was comparable to the mean number of ablations in the UK Registry over 12 months (2.5) 24 .

Our study does have some limitations. First, it was not powered to detect a difference in stricture rate between the groups. Second, we only assessed short-term outcomes of RFA. Although we applied a rigorous protocol with two consecutive biopsies to confirm CR-D or CR-IM, overall follow-up was relatively short, therefore, our data are insufficient to provide information on the durability of RFA comparing these two protocols. However, the long-term durability of RFA in one of the two centers has been reported elsewhere 7 . Third, RFA was performed in tertiary institutions with high-volume caseload and experienced endoscopists, therefore, the results of this study may not necessarily reflect the practice in small-volume centers. Finally, the observed rate of strictures within the standard protocol group (1.4 %) was lower than that observed in the previous prospective and registry studies. While we are unable to explain this low stricture rate, we cannot exclude that it may have had an impact on the difference observed between the two groups.

## Conclusion

In conclusion, we showed that the simplified RFA protocol for the whole treatment pathway, including both circumferential and focal ablations, is just as effective as the standard protocol. The added benefit of fewer introductions of the device, shorter procedural time, and likely less discomfort for patients makes it favorable. However, the higher rate of strictures needs to be considered when making decisions on an individual basis, especially in patients with higher pretreatment probability of stricture, such as those with esophageal stenosis from previous EMR.
